# T1 Mapping in Cardiovascular Magnetic Resonance—A Marker of Diffuse Myocardial Fibrosis in Patients Undergoing Hematopoietic Stem Cell Transplantation

**DOI:** 10.3390/jpm14040412

**Published:** 2024-04-13

**Authors:** Audrone Vaitiekiene, Migle Kulboke, Monika Bieseviciene, Antanas Jankauskas, Agne Bartnykaite, Diana Rinkuniene, Igne Strazdiene, Emilija Lidziute, Darija Jankauskaite, Ignas Gaidamavicius, Paulius Bucius, Tomas Lapinskas, Rolandas Gerbutavicius, Elona Juozaityte, Jolanta Justina Vaskelyte, Domas Vaitiekus, Gintare Sakalyte

**Affiliations:** 1 Department of Cardiology, Medical Academy, Lithuanian University of Health Sciences, 44307 Kaunas, Lithuania; 2 Department of Oncology and Hematology, Medical Academy, Lithuanian University of Health Sciences, 44307 Kaunas, Lithuania; 3 Oncology Institute, Lithuanian University of Health Sciences, 50161 Kaunas, Lithuania; 4 Department of Radiology, Medical Academy, Lithuanian University of Health Sciences, 44307 Kaunas, Lithuania; 5 Institute of Cardiology, Lithuanian University of Health Sciences, 44307 Kaunas, Lithuania; 6 Oncology Research Laboratory, Oncology Institute, Lithuanian University of Health Sciences, 50161 Kaunas, Lithuania; 7 Institute of Physiology and Pharmacology, Medical Academy, Lithuanian University of Health Sciences, 44307 Kaunas, Lithuania; 8 Medical Academy, Lithuanian University of Health Sciences, 44307 Kaunas, Lithuania

**Keywords:** hematopoietic stem cell transplantation, cardiotoxicity, cardiovascular magnetic resonance, T1 mapping, cardio-oncology

## Abstract

**Introduction:** Hematopoietic stem cell transplantation (HSCT) recipients are at increased risk of cardiovascular diseases. In our study, we aimed to find subclinical changes in myocardial tissue after HSCT with the help of cardiovascular magnetic resonance (CMR) tissue imaging techniques. **Methods:** The data of 44 patients undergoing autologous and allogeneic HSCT in the Hospital of Lithuanian University of Health Sciences Kaunas Clinics from October 2021 to February 2023 were analyzed. Bioethics approval for the prospective study was obtained (No BE-2-96). CMR was performed two times: before enrolling for the HSCT procedure (before starting mobilization chemotherapy for autologous HSCT and before starting the conditioning regimen for allogeneic HSCT) and 12 ± 1 months after HSCT. LV end-diastolic volume, LV end-systolic volume, LV mass and values indexed to body surface area (BSA), and LV ejection fraction were calculated. T1 and T2 mapping values were measured. **Results:** There was a statistically significant change in T1 mapping values. Before HSCT, mean T1 mapping was 1226.13 ± 39.74 ms, and after HSCT, it was 1248.70 ± 41.07 ms (*p* = 0.01). The other parameters did not differ significantly. **Conclusions:** Increases in T1 mapping values following HSCT can show the progress of diffuse myocardial fibrosis and may reflect subclinical injury. T2 mapping values remain the same and do not show edema and active inflammation processes at 12 months after HSCT.

## 1. Introduction

Hematopoietic stem cell transplantation (HSCT) can be a potentially curative procedure for various malignant hematologic diseases and some solid tumors, where high dose chemotherapy is used for treatment [1,2]. HSCT survival has significantly improved over the last few decades [3]. Although HSCT is quite often followed by short- or long-term complications, improvements in transplantation techniques and supportive strategies have markedly decreased treatment-related mortality, and therefore, the number of HSCT survivors is expected to exceed half a million by 2030 [4]. 

The main characteristics of hematopoietic stem cells (HSC) are their capacity to self-renew and multipotency—the ability to generate all mature hematopoietic cell types by inducing a proliferation and differentiation program driven by the expression of sets of transcription factors. Because of these properties, multilineage hematopoiesis can be maintained throughout the whole life of an individual. HSC with properties of long-term hematopoietic reconstitution can be identified from bone marrow or chemotherapy and/or granulocyte colony-stimulating factors (G-CSF) mobilized peripheral blood or umbilical cord blood [5,6,7]. HSCs can be used for therapeutic purposes: the transplantation of autologous or allogeneic HSCs for the reconstitution of hematopoiesis in patients after intensive chemo- or radiotherapy when treating malignant disease; allogeneic HSCs in patients with the failure of bone marrow; or gene therapy via inserting normal gene copies into genetically defective stem cells, which could then be transplanted [8,9,10]. 

HSCT is a multi-step procedure that includes the collection of hematopoietic stem cells, the treatment of the patient’s main disease with a conditioning regimen followed by the infusion of HSCs, and the subsequent evolvement of a new hematopoietic and immune system [11]. Two fundamentally different types of HSCT are characterized by the source of stem cells: autologous hematopoietic stem cell transplantation, where the stem cells are collected from the recipient him/herself during mobilization procedure and are later reinfused, and allogeneic hematopoietic stem cell transplantation, where the cells are taken from a different person (donor), who can be related or unrelated to the patient [11,12]. The major benefit of autologous HSCT is achieved by the effects of the conditioning treatment. The infusion of hematopoietic stem cells allows the delivery of toxic therapies for the treatment of main disease, which would otherwise result in prolonged myelosuppression and a high risk of complications, including fatal outcomes [13]. Allogeneic HSCT can cure certain hematologic malignancies through several mechanisms: high doses of chemotherapy and radiation that a patient receives before the infusion of the HSC graft (conditioning or preparatory regimen) and the immunity-mediated graft versus disease (GVD) reaction [14].

However, HSCT is associated with some serious complications, starting with different grade infections and sepsis. A specific and most limiting complication for allogeneic HSCT is graft versus host disease (GvHD), an immune rejection to host tissues mediated by donor lymphocytes which results in a skin rash, diarrhea, and liver disease. This condition can become chronic and produce a systemic sclerosis-like illness [12]. Allogeneic hematopoietic stem cell transplant-related mortality can be as high as over 30% at 1 year post-transplant [15]. Organ dysfunctions (cardiac, pulmonary, endocrine, and musculoskeletal), infertility, and secondary cancers are significantly more prevalent among allogeneic hematopoietic stem cell transplantation survivors than the general population [12]. Moreover, there is a growing awareness of the negative effects of HSCT-related therapies on the cardiovascular system, and HSCT recipients are at increased risk of cardiovascular disease later in life. Studies state that cardiovascular disease risk is at least fourfold higher than in general population [16,17]. According to the literature, among all complications related to HSCT, cardiovascular complications account for around 10–16.84% for both allogeneic and autologous transplantation, but they decrease the quality of life for long-term survivors and lead to a high mortality rate [18,19]. The most common complications are arrhythmias (mainly atrial fibrillation, atrial flutter, and supraventricular tachycardia), congestive heart failure, pericardial effusion including cardiac tamponade, ischemic heart disease, and rarely ventricular arrhythmias. Long-term complications can be crucial for patient survival [17,20,21].

According to the recently published guidelines of cardio-oncology developed by the European Society of Cardiology (ESC), together the European Hematology Association (EHA), the European Society for Therapeutic Radiology and Oncology (ESTRO), and the International Cardio-Oncology Society (IC-OS), cardiotoxicity risk can be classified according to cardiovascular risk factors, pre-existing cardiovascular morbidities, previous cardiotoxic anticancer treatment effects, and the type of HSCT: allogeneic versus autologous [11,22].

Cardiac adverse events can be related to different components of HSCT such as ablative therapy, including total body radiation combined with a multi-drug conditioning regimen. Most drugs used for mobilization or conditioning, including cyclophosphamide, cytarabine, carmustine, and melphalan, are associated with significant toxicity. Additionally, effects attributed to dimethylsulfoxide used to preserve stem cells are also thought to contribute to cardiac events. Moreover, monoclonal antibodies and other targeted therapies used before and after HSCT are also associated with many cardiac side effects. Cardiac complications may also arise as consequences of other HSCT-related comorbidities such as graft versus host disease, sepsis, thrombotic microangiopathy, or hepatic veno-occlusive disease [23]. 

What is more, not only can clinically obvious cardiovascular complications occur but subclinical damage may also be noticed even more often, and the exact frequency is not known. Subclinical damage can be related to clinical cardiovascular diseases later in life, so it is important to detect early changes in order to prevent subsequent complications. Pre-transplant or HSCT-related cardiotoxic treatment and the HSCT-related comorbidities mentioned above can cause myocyte cell death with reactive interstitial fibrosis [24,25]. Myocardial diffuse fibrosis is considered to reflect subclinical disease before cardiac dysfunction manifests in different types of cardiomyopathies [26]. 

There are numerous studies trying to identify different imaging markers which could indicate subclinical changes in the myocardium [27,28]. This would support the detection of high-risk groups of patients for closer monitoring or preventive treatment [16]. Cardiovascular magnetic resonance (CMR) is a non-invasive comprehensive imaging modality that provides not only precise anatomical information but also tissue characteristics and cardiometabolic assessment, which leads to its increased use in the early identification of cardiotoxicity [29]. CMR has the ability to depict changes at the tissue level, which could be a reflection of physiology and pathophysiology, in addition to data regarding left ventricular (LV) function (ejection fraction (EF), strain), volume, and mass [30]. Myocardial tissue characterization techniques, including gadolinium enhancement sequences and mapping techniques, enable the detection of myocardial edema, inflammation, and fibrosis [29]. There are numerous studies where CMR was analyzed for the comprehensive evaluation of the subclinical changes in the cardiovascular system after HSCT [16,29].

T1 and T2 mapping are parametric quantitative sequences which provide tissue-specific T1 and T2 values. No contrast agent is needed to obtain these sequences. The comparison of quantified myocardial tissue parameters can be performed. Representative myocardial pathologies leading to T1 changes involves mainly diffuse myocardial fibrosis, and T1 prolongation can also be observed in the presence of edema; inflammation; infiltrative diseases, such as amyloidosis; and Fabry disease. T2 relaxation time is also used to distinguish between normal and abnormal myocardial tissues. The increase in the water content of myocardial tissues causes longer T2 relaxation times. Therefore, myocardial edema is the main pathology responsible for variation in T2 values [31].

In this study, we aimed to evaluate changes in LV volumes and function and to find subclinical changes in myocardial tissue after HSCT with the help of CMR tissue imaging techniques.

## 2. Materials and Methods

The study was performed prospectively. The data of 44 patients undergoing autologous and allogeneic HSCT at the Department of Oncology and Hematology in the Hospital of Lithuanian University of Health Sciences Kaunas Clinics were analyzed. The study period lasted from October 2021 to February 2023. The study protocol conforms to the ethical guidelines of the 1975 Declaration of Helsinki, and approval of bioethics for the prospective study was obtained (No BE-2-96). All the patients have read and signed the informed consent form. 

The inclusion criteria were as follows: (1) written consent to participate in the study and (2) patients over the age of 18 years who were scheduled for autologous or allogeneic HSCT for various reasons. Exclusion criteria were: (1) contraindications to CMR, for example non-MR conditional implants or claustrophobia, and (2) patient’s refusal to participate at any time in the study.

CMR for each patient was performed and evaluated twice: before enrolling for HSCT procedure (for patients undergoing autologous HSCT—before starting mobilization chemotherapy; and for patients undergoing allogeneic HSCT—before starting the conditioning regimen) and 12 ± 1 months after HSCT. The aim was to evaluate the changes in LV volumes, mass, and function and the parametric T1 and T2 mapping.

Patients underwent autologous or allogeneic HSCT for various reasons. The distribution of diseases and the type of transplantation are described in detail in Table 1. Hematologic malignancies were treated according to the local institution treatment protocols based on international guidelines. Hematopoietic stem cell harvesting for autologous HSCT was performed with chemotherapy and granulocyte colony stimulating factors (G-CSF)—chemo-mobilization. Multiple myeloma patients were given cyclophosphamide at 3000 mg/m^2^; mantle cell lymphoma patients were given rituximab at 375 mg/m^2^ and cytarabine at 4 g/m^2^; Hodgkin’s lymphoma patients were given cisplatin at 30 mg/m^2^ and cytarabine at 3.5 g/m^2^; primary central nervous system (PCNS) diffuse large B cell lymphoma patients were given cytarabine and thiotepa at different doses and rituximab at 375 mg/m^2^; a NK/T-cell lymphoma patient underwent the CHOEP (cyclophosphamide, doxorubicin, vincristine, etoposide, and prednisone) scheme; and an Ewing sarcoma patient underwent an IE scheme (ifosfamide with etoposide). All patients scheduled for autologous HSCT underwent apheresis procedures. Hematopoietic stem cells from peripheral blood were collected using Fresenius Kabi COM.TEC apheresis system. The cells were then cryopreserved in a solution of 10% DMSO and autologous plasma. Cryopreservation was performed in a controlled-rate freezer with liquid nitrogen (Consarctic equipment). Conditioning was performed as follows: multiple myeloma patients with melphalan 200 mg/m^2^, lymphomas with BEAM (carmustine, etoposide, cytarabine, melphalan) protocol, primary central nervous system (PCNS) diffuse large B cell lymphoma—thiotepa and BCNU. Hematopoietic stem cells for patients undergoing allogeneic transplantation were isolated from donors via apheresis procedures. All donors were unrelated, with an HLA match of 10/10. CMV, ABO blood groups, and sex were the best available options chosen by local protocols for donor selection criteria. Peripheral stem cells were harvested by G-CSF stimulation and apheresis procedure for all patients. Grafts were used for a maximum 48 h after the apheresis, and no grafts were frozen. All patients undergoing allogeneic HSCT received reduced intensity conditioning (RIC) with fludarabine and busulfan. 

All patients filled in a survey regarding cardiovascular risk factors (arterial hypertension, smoking, cardiovascular family history, dyslipidemia, and diabetes mellitus). Information regarding coronary artery disease (CAD) and cardiovascular medication was obtained during the interview and double-checked against medical records. Arterial hypertension was diagnosed, and the grade of arterial hypertension was established according to the European Society of Cardiology and European Society of Hypertension guidelines, published in 2018. Arterial hypertension was diagnosed if systolic arterial blood pressure was ≥140 mmHg and/or diastolic arterial blood pressure was ≥90 mmHg [32]. A family history of early coronary artery disease was defined as a cardiovascular event (stroke, myocardial infarction, revascularization procedures) or cardiovascular death in first-degree relatives (men ≤ 55 years old; women ≤ 65 years old). 

### 2.1. CMR Acquisition and Analysis

All study participants underwent 3T CMR with an 18-channel cardiac coil (MAGNETOM Skyra, Siemens Healthcare, Erlangen, Germany). Standard electrocardiographic (ECG) triggered two-, three-, and four-chamber sequences, and short-axis cine-balanced steady-state free precession sequences were performed. The quantitative analysis of LV end-diastolic volume (LV EDV), LV end-systolic volume (LV ESV), LV mass, and all these values indexed to body surface area (BSA); also, LV EFs were analyzed and calculated using Medis Suite 3.2 (Leiden, The Netherlands). The LV endo- and epi-contours were outlined manually in cine short axis views in the end-diastolic and end-systolic phases, with the exclusion of the LV papillary muscles. Global LV systolic function was assessed by calculating the difference between the LV end-diastolic and end-systolic volumes, divided by the end-diastolic volume.

LV EF = (LVEDV – LVESV)/LVEDV

**Figure 1:**
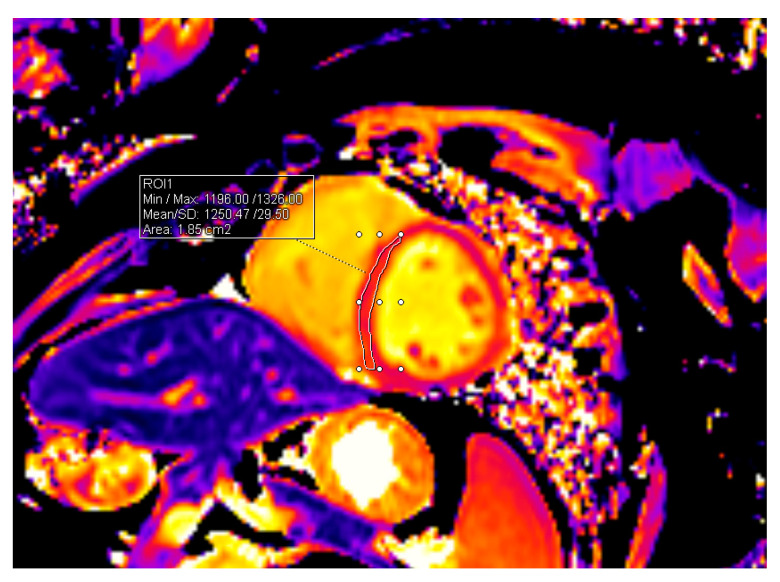
Measurement of T1 value in the midventricular slice.

**Figure 2:**
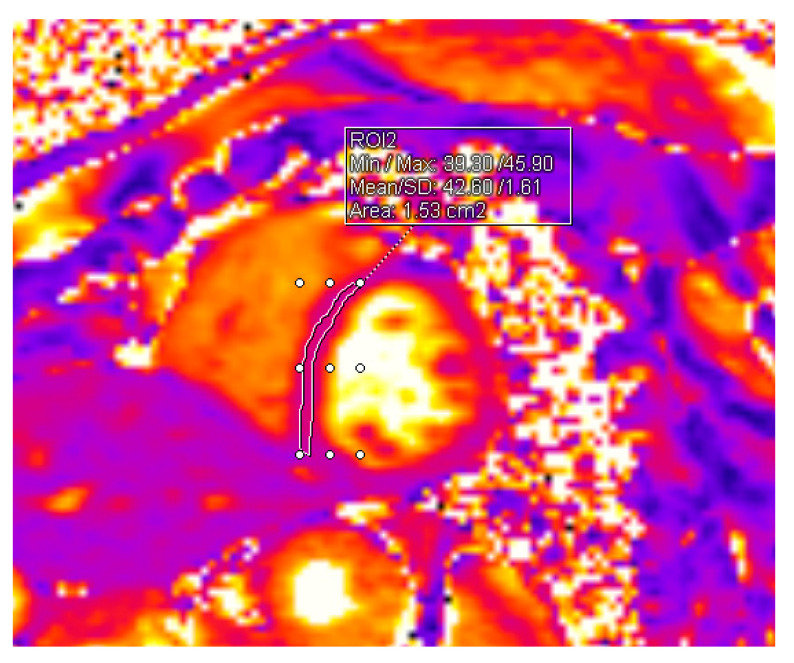
Measurement of T2 value in the midventricular slice.

T1 and T2 mapping sequences were obtained in end-diastole in short-axis orientation in three slices (basal, midventricular, and apical). For myocardial T1 mapping, MOLLI (modified Look-Locker inversion) recovery acquisition scheme with motion correction (MOCO) was applied. T2 mapping was performed using T2-prepared balanced steady-state free precession sequence. T1 and T2 relaxation times were analyzed using a dedicated Syngo.via postprocessing system. The interventricular septum was outlined in all three slices (basal, midventricular, and apical) and the averages of all three measurements were obtained. Baseline mapping values before the beginning of HSCT were compared to values acquired 12 months after HSCT. The methods of measurements are shown in Figure 1 and Figure 2.

### 2.2. Statistical Analysis

Statistical analysis was performed with the help of IBM SPSS Statistics 20.0. Qualitative data are presented as absolute values (N) and percentages (%). The normality of data distribution was evaluated with the Kolmogorov–Smirnov test, and quantitative parameters are given as average ± standard deviation or median (minimum–maximum). Variables were compared using Student’s *t*-test. A statistically significant difference was considered when *p* < 0.05.

## 3. Results

The 44 patients comprised 24 men (54.5%) and 20 women (45.5%). The median age was 61 years, ranging from 18 to 74 years. Thirty-nine patients (88.6%) underwent autologous HSCT and five patients (11.4%) underwent allogeneic HSCT. HSCT was performed for various reasons. The main demographic characteristics of the patients and the main diseases for which HSCT was applied are listed in Table 1.

The distribution of different cardiovascular risk factors among patients is presented in Table 2. Four (9.1%) patients had coronary artery disease, fifteen (34.1%) patients had arterial hypertension, three (6.8%) patients had diabetes mellitus, eight (18.2%) patients had positive family history of early CAD, thirty-one (70.5%) patients had dyslipidemia, and zero (0%) patients were current smokers.

Patients with CAD and arterial hypertension tended to have a larger LV mass and LV mass index at the beginning of the study than patients without CAD and arterial hypertension. In CAD patients, LV mass before HSCT was 144.07 ± 29.45 g vs. 105.74 ± 26.31 g in patients with no CAD (*p* = 0.009). LV mass index was 67.54 ± 13.51 g/m^2^ vs. 55.29 ± 10.46 g/m^2^, respectively (*p* = 0.035). In patients with arterial hypertension LV mass before HSCT was 122.51 ± 32.05 g vs. 102.35 ± 24.30 g in patients with no arterial hypertension (*p* = 0.044). LV mass index was 60.64 ± 13.38 g/m^2^ vs. 54.21 ±9.35 g/m^2^, respectively (*p* = 0.041).

Two patients (4.5%) had clinical cardiovascular symptoms—the onset of supraventricular tachycardia during the 12-month observation period. No other clinically relevant signs or symptoms of cardiotoxicity—heart failure, pericardial effusion, new onset or worsening of arterial hypertension, or acute ischemic syndromes—were noticed.

There was a statistically significant change in T1 mapping value. Before HSCT, mean T1 mapping was 1223.13 ± 39.74 ms, and 12 months after HSCT, it was 1248.70 ± 41.07 ms (*p* = 0.010). No statistically significant change in T2 mapping values was noticed—T2 mapping mean value before HSCT was 38.91 ± 2.07 ms, and 12 months after HSCT, it was 38.53 ± 2.11 ms (*p* = 0.430). The change in T1 mapping was significant in all patients, independent of cardiovascular risk factors. The changes are listed in Table 3.

No statistically significant change in LV volumes, systolic function, or mass was observed. The mean LV EDV before HSCT was 142.45 ± 35.62 mL, and after HSCT, it was 145.29 ± 36.36 mL (*p* = 0.713). The mean indexed LV EDV before HSCT was 74.33 ± 15.23 mL/m^2^ and after HSCT, it was 75.64 ± 15.10 mL/m^2^ (*p* = 0.686). The mean LV ESV before HSCT was 60.82 ± 18.36 mL, and after HSCT, it was 60.15 ± 17.17 mL (*p* = 0.860), and the LV ESVi was 31.07 ± 8.36 mL/m^2^ vs. 31.55 ± 8.61 mL/m^2^, respectively (*p* = 0.791). The mean LV EF before HSCT was 58.10 ± 7.77%, and after HSCT, it was 58.58 ± 7.08% (*p* = 0.761). Th mean LV mass before HSCT was 109,22 ± 28,51 g, and after HSCT, it was 107.81 ± 29.44 g (*p* = 0.819), and the mean LV mass index was 56.40 ± 11.17 g/m^2^ vs. 56.37 ± 12.92 g/m^2^, respectively (*p* = 0.991). The changes are summarized in Table 4.

## 4. Discussion

In this prospective study, we aimed to evaluate subclinical changes in LV function, volumes, mass, and mainly in the myocardial tissue, with the help of CMR in a one-year period after HSCT, irrespective of the type of HSCT. 

Our results showed no significant change in LV volumes, mass, systolic function, and T2 mapping, a marker of myocardial edema. T1 mapping had statistically significantly higher values in one year after HSCT. This could prompt an idea that the HSCT procedure is related to the increase in diffuse myocardial fibrosis. 

T1 and T2 mapping techniques in cardiovascular magnetic resonance have been validated histologically in a study with rat models. Park et al. analyzed the myocardial injuries of rats that were injected with doxorubicin. T1 and T2 mapping and extracellular volume (ECV) values were calculated, and myocardial biopsy was performed. The study showed that T1 and T2 measures correlate to histopathologic changes that represent myocardial injury, in particular, the interstitial fibrosis, inflammation, and edema of myocardial biopsy with anthracycline-induced cardiotoxicity. T1 mapping and ECV showed the highest correlations with histopathologic changes [33].

In our study, LV EDV, LV EDVi, LV ESV, LV ESVi, and LV EF did not change statistically significantly in 12 months after HSCT. The results of our study are consistent with findings from other studies. Rotz and colleagues analyzed the echocardiographic parameters of 95 children and young adults. Echocardiography was performed before and 1–6 years after HSCT, and LV EF, as well as global longitudinal and global circumferential strains, were analyzed. The results of the study show that after HSCT, LV EF, as well as global longitudinal and global circumferential strains, remained unchanged from the baseline [34].

Despite the fact that LV EDV and EF did not change statistically significantly, our results show that T1 mapping values that could resemble the progress of a diffuse fibrosis increase. Changes in native T1 values in patients after HSCT compared to healthy individuals have also been revealed in several other studies. Paiman et al. analyzed the late effects of pediatric HSCT on LV function, aortic stiffness, and myocardial tissue characteristics. The study design was different—they analyzed 16 HSCT childhood survivors (14.8 ± 5 years after HSCT) and compared them to 16 healthy controls. They noticed a trend towards a lower LV EF in HSCT survivors compared to healthy controls, but the difference was not statistically significant (54 ± 6 vs. 58 ± 5%, *p* = 0.055). Native T1 (1211 ± 36 vs. 1227 ± 28 ms, *p* = 0.158) also tended to be higher in HSCT survivors than in healthy controls, but statistical significance was not reached. Moreover, the method of comparison was different—they compared the values to healthy controls and not to the baseline values. Also, the sample was very small [16].

There are more studies analyzing cardiotoxicity with the help of T1 mapping techniques. Most studies analyze anthracycline-induced cardiotoxicity. A study conducted by Jordan et al. concluded that the elevation in myocardial T1 mapping and ECV values was noticed three years after anthracycline-based chemotherapy independently of the main underlying disease or cardiovascular comorbidities [35]. The other study analyzed changes in T1 mapping in childhood cancer survivors who received anthracycline-based chemotherapy and had normal systolic function. CMR evaluation was performed 2.5 to 26.9 years after anthracycline exposure. Pre-contrast T1 values were higher in childhood cancer survivors than in healthy controls, although the difference did not reach statistical significance [36].

In another study, T1 mapping and ECV were found to be early tissue markers of ventricular remodeling, representing diffuse fibrosis in children with normal LV EF post anthracycline therapy [37].

We did not identify a significant change in T2 mapping values. The increase in T2 mapping parameters resemble the features of edema. Our follow-up was conducted 12 months after the HSCT process, so active inflammation and edema in the myocardium were not expected at this time point.

The strength of our study is that it was performed prospectively. Therefore, we can compare baseline and follow-up values. The other studies mostly compared patients after HSCT or chemotherapy treatment to healthy controls. We did not notice any statistically significant impact of cardiovascular risk factors, main disease, and treatment regimens on T1 mapping, and the values increased in all patients. The biggest change was noticed in patients with diabetes mellitus (T mapping before HSCT was 1231.79 ± 11.35 ms, and it was 1277.14 ± 47.68 ms 12 months after HSCT) but the number of the patients was very small—only three patients. This could lead to a hypothesis that patients with diabetes mellitus tend to have more extensive diffuse myocardial fibrosis following HSCT, but further investigation and a bigger sample is needed.

### Limitations

Due to the relatively small sample, the influence of different cardiovascular risk factors, main disease, and treatment regimens on diffuse myocardial fibrosis could not be defined. Further investigations with larger numbers of patients are needed. 

## 5. Conclusions

Native T1 mapping values following HSCT increased compared to baseline values, which could resemble the progress of diffuse myocardial fibrosis and may reflect subclinical injury before clinical cardiovascular complications manifest. T2 mapping values remain the same and do not show edema or active inflammation processes at 12 months after HSCT. 

## Figures and Tables

**Table 1:** Characteristics of patients. HSCT: hematopoietic stem cell transplantation; PCNS: primary central nervous system; NK: natural killer.

**Sex**	
Male, *n* (%)	24 (54.5)
Female, *n* (%)	20 (45.5)
**Age**, years (median (minimum–maximum))	61 (18–74)
**Autologous HSCT**, n (%)	39 (88.6)
**Main disease**	
Multiple Myeloma, *n* (%)	27 (69.2)
PCNS diffuse large B cell lymphoma, *n* (%)	4 (10.3)
Mantle cell lymphoma, *n* (%)	4 (10.3)
Hodgkin’s lymphoma, *n* (%)	2 (5.1)
NK/T-cell lymphoma, *n* (%)	1 (2.6)
Ewing sarcoma, *n* (%)	1 (2.6)
**Allogeneic HSCT**, *n* (%)	5 (11.4)
**Main disease**	
Acute myeloid leukemia, *n* (%)	4 (80.0)
Acute myelomonocytic leukemia, *n* (%)	1 (20.0)

**Table 2:** Distribution of cardiovascular risk factors among the HSCT patients. CAD—coronary artery disease.

Cardiovascular Risk Factors	*n* (%)
CAD	4 (9.1)
Arterial hypertension	15 (34.1)
Diabetes mellitus	3 (6.8)
Family history of CAD	8 (18.2)
Dyslipidemia	31 (70.5)
Currently smoking	0 (0)

**Table 3:** Mean values of T1 mapping before and after HSCT in patients with cardiovascular risk factors. CAD—coronary artery disease.

Cardiovascular Risk Factor	Mean T1 Values before HSCT	Mean T1 Values after HSCT	No. of Patients
CAD	1212.54 ± 17.62	1235.27 ± 17.82	4
Arterial hypertension	1235.52 ± 42.24	1257.07 ± 50.14	15
Diabetes mellitus	1231.79 ± 11.35	1277.14 ± 47.68	3
Family history of CAD	1224.16 ± 34.53	1250.08 ± 43.54	8
Dyslipidemia	1227.49 ± 35.75	1245.69 ± 43.67	31

**Table 4:** The change in CMR parameters before and after HSCT. LV EDV—left ventricular end-diastolic diameter; LV EDVi—indexed left ventricular end-diastolic diameter; LV ESV—left ventricular end-systolic diameter; LV ESVi—indexed left ventricular end-systolic diameter; LV EF—left ventricular ejection fraction; LV—left ventricle.

CMR Values	Before HSCT (Mean ± SD)	After HSCT (Mean ± SD)	*p*
LV EDV, mL	142.45 ± 35.62	145.29 ± 36.36	0.713
LV EDVi, mL/m^2^	74.33 ± 15.23	75.64 ± 15.10	0.686
LV ESV, mL	60.82 ± 18.36	60.15 ± 17.17	0.860
LV ESVi, mL/m^2^	31.07 ± 8.36	31.55 ± 8.61	0.791
LV EF, %	58.10 ± 7.77	58.58 ± 7.08	0.761
LV mass, g	109.22 ± 28.51	107.81 ± 29.44	0.819
LV mass index, g/m^2^	56.40 ± 11.17	56.37 ± 12.92	0.991
T1 mapping value, ms	1226.13 ± 39.74	1248.70 ± 41.07	**0.010**
T2 mapping value, ms	38.91 ± 2.14	38.53 ± 2.14	0.412

## Data Availability

The data presented in this study are available on request from the corresponding author.
